# Crosslinked Recombinant-Ara h 1 Catalyzed by Microbial Transglutaminase: Preparation, Structural Characterization and Allergic Assessment

**DOI:** 10.3390/foods9101508

**Published:** 2020-10-21

**Authors:** Yang Tian, Chenglong Liu, Wentong Xue, Zhongfu Wang

**Affiliations:** 1 College of Food Science and Technology, Northwest University, Xi’an 710069, China; tiany1030@nwu.edu.cn (Y.T.); wangzhf@nwu.edu.cn (Z.W.); 2 Research Center for Glycobiology and Glycotechnology, College of Food Science and Technology, Northwest University, Xi’an 710069, China; 3 College of Food Science and Nutritional Engineering, China Agricultural University, Beijing 100083, China; CLL@cau.edu.cn

**Keywords:** peanut allergy, cross linking, structural features, immunoreactivity changes

## Abstract

As the one of the major allergens in peanut, the allergenicity of Ara h 1 is influenced by its intrinsic structure, which can be modified by different processing. However, molecular information in this modification has not been clarified to date. Here, we detected the influence of microbial transglutaminase (MTG) catalyzed cross-linking on the recombinant peanut protein Ara h 1 (rAra h 1). Electrophoresis and spectroscopic methods were used to analysis the structural changes. The immunoreactivity alterations were characterized by enzyme linked immunosorbent assay (ELISA), immunoblotting and degranulation test. Structural features of cross-linked rAra h 1 varied at different reaction stages. Hydrogen bonds and disulfide bonds were the main molecular forces in polymers induced by heating and reducing. In MTG-catalyzed cross-linking, ε-(γ-glutamyl) lysine isopeptide bonds were formed, thus inducing a relatively stable structure in polymers. MTG catalyzed cross-linking could modestly but significantly reduce the immunoreactivity of rAra h 1. Decreased content of conserved secondary structures led to a loss of protection of linear epitopes. Besides, the reduced surface hydrophobic index and increased steric hindrance of rAra h 1 made it more difficult to bind with antibodies, thus hindering the subsequent allergic reaction.

## 1. Introduction

Food allergy has become a severe health problem worldwide. In 2018, approximately 10% of people were reported to be affected by food allergy [1]; the prevalence in children (4–6%) is slightly higher than which in adults (1–3%) [2]. A short list of foods containing peanut, tree nuts, fish, shellfish, egg, milk, wheat, soy, and seed are responsible for most of the serious disease burden. Peanut allergy has attracted the most attention in the food allergy research area, for the allergy caused by peanut is relatively common, typically permanent, and often severe. Even traces of peanut protein (1.25–2.50 mg) could trigger the allergy reactions [3], and then cause undesired symptoms like urticarial, diarrhea, and bronchospasm [4]. The composition and structure of peanut proteins has been thoroughly explored during the last 40 years, and 17 allergens have been identified [5]. As one of the major allergens in peanut, Ara h 1 accounts for 12% of the total protein content, and could be recognized by 55–95% of peanut-allergic patients’ serum in Britain [6]. Approximately 90% of peanut-allergic patients have antibodies against Ara h 1 [7,8]. It is practically significant to establish effective strategies to lower the risk of peanut allergy. To achieve this goal, researchers have targeted the peanut major allergens and performed different treatments.

Processing, which has been tested to reduce the immunoreactivity of food allergens, can be classified into thermal treatment [9] (like boiling, roasting, frying), ultrasound [10], autoclaving [11], acid or enzymatic hydrolysis [12], germination [13], fermentation [14], electric field treatment [15], enzyme-mediated cross-linking [16], etc. Solubility changes, structural alterations and the following allergenicity changes in allergens were easily induced by processing, while the extent of the effects was influenced both by processing procedures and the biochemical properties of the treated allergen. For example, the immunoreactivity of Ara h 1 was reduced after boiling, for the allergen was attacked by water molecules, and aggregates with a significantly lowed Immunoglobulin E (IgE) binding ability were subsequently formed [17,18,19]. The immunogenicity of recombinant Ara h 1 (rAra h 1) glycosylated by glucosamine was significantly decreased, because the linear and conformational epitopes were destructed by molecular modifications [16]. On the contrary, dendritic cells had a higher uptake of the glycated allergens like Ara h 2 [20], which then caused an increased sensitivity of the mentioned allergen. At present, those processing cannot be proved to be effectively eliminate the allergenicity of peanut (or one specific peanut allergen) completely, and so developing a new approach which could reduce the allergenicity of peanut allergens is important for public health.

Enzymatically catalyzed cross-linking is an innovative processing strategy to modify the food allergen and thus modulate the sensitivity. Transglutaminase (TGase, EC 2.3.2.13) has been extensively utilized in modern food industries like meat packing and Tofu production [21], for it could catalyze acyl transfer reactions between the γ-carboxyamide of glutamine residues (acyl-donors) and various primary amines (acyl acceptors) [22]. The ε-(γ-glutamyl) lysine isopeptide bonds are formed when the ε-amino groups of lysine residues act as acyl acceptors [23], and cross-linking between protein molecules would subsequently happen. Recently, there is some research beginning to use microbial transglutaminase (MTG) in desensitization research. For example, cross-linking catalyzed by MTG have been proved to effectively lower the allergenicity of Ara h 2, for the linear allergen epitopes were destroyed during the reaction [24]. It is also worth noting that DL-dithiothreitol (DTT) were added in the reaction system to release the inner glutamine and lysine residues. Actually, adjuvants like caffeic acid, tannic acid, catechol, phenol and phloretic acid could act as assistant mediators in cross-linking and have been widely used in tyrosinase-aided or polyphenol-oxidase-catalyzed reactions [25,26,27]. The addition of reductant or phenolic acid in cross-linking could help to destabilize the protein by breaking chemical bonds and exposing residues (like glutamine and lysine) to the protein surface, thus accelerating the reaction. According to the sequence data of Ara h 1 [28], seven cystines existed in the amino acid structure of rAra h 1. There is a great possibility that disulfide bonds were formed between cystines. In this research, DTT was chosen to break the disulfide bonds, and thus decrease the immunoreactivity of rAra h 1.

To date, the detailed structural changes and antigenicity alterations to Ara h 1 during the MTG-catalyzed crosslinking have not been clarified. Besides this, glutamine is contained in about half of the linear epitopes in Ara h 1 [16], therefore there is potential that MTG-catalyzed cross-linking would alter the sensitivity of Ara h 1. The structural features and immunoreactivity of recombinant Ara h 1 (rAra h 1) are similar to natural Ara h 1, and rAra h 1 is easier to purify when compared with the parental Ara h 1 [17]. In sum, we aimed to explore the optimal conditions which were used for the rAra h 1 cross-linking catalyzed by MTG, and define the effect of cross-linking on the structure and allergenicity of rAra h 1.

## 2. Materials and Methods

### 2.1. Material and Reagents

The coding DNA of full-length Ara h 1 [28], adding 6 × his tag, was cloned into a pET-32a expression vector by Shenggong biology company (Shanghai, China). Recombinant Ara h 1 (rAra h 1) was produced by *Escherichia coli BL21(DE3) pLysS* and purified as described [17]. Bovine serum albumin (BSA), microbial transglutaminase (MTG), 3,3′,5,5′-tetramethylbenzidine (TMB), isopropyl-β-D-thiogalactopyranoside (IPTG), 4-nitrophenyl-N-acetyl-β-D-glucosaminide (PNAG), 1-anilino-8-naphthalene-sulfonate (ANS), and peroxidase (HRP)-labeled goat anti-human IgE were purchased from Sigma Chemical Co. (St Louis, MO, USA). Precast 4–20% electrophoresis gel kits, loading buffer, DL-dithiothreitol (DTT), BCA Protein Assay Kit, and peroxidase (HRP)-labeled goat anti-rabbit IgG were obtained from Solarbio Co. (Beijing, China). The standard protein marker was obtained from TransGen Biotech Company (Beijing, China). Rabbit anti-Ara h 1 antibody was kindly provided by the university of Manchester. Enhanced chemiluminescence (ECL) kit for immunoblotting and ImmunoCAP assay kit were purchased from Beyotime Co. (Shanghai, China).

### 2.2. Human Sera

Sera from twelve peanut allergic patients were provided by the Affiliated Hospitals of China Agricultural University (Beijing, China) and Northwest University (Xi’an, China). All the patients were confirmed to be allergic to peanut by a clinical team according to physical examination, skin prick testing, and objective manifestations observed after peanut ingestion (Table S1). The IgE levels were measured by ImmunoCAP assay kit according to the manufacturer’s instructions. All subjects gave their informed consent for inclusion before they participated in the study. The study was conducted in accordance with the Declaration of Helsinki, and the protocol was approved by the Ethics Committee of China Agricultural University. The ethical approval can be found in the supporting file.

### 2.3. Preparation of Cross-Linked rAra h 1

rAra h 1 was ultra-filtrated and freeze-dried. Protein and MTG powder were dissolved in Tris-HCl (50 mM, pH 7.5). The working concentrations of rAra h 1 and MTG are 1.0 mg/mL and 1 U/mL separately. Methods used in the cross-linking from Wu were modified as follows [24].

For the cross-linking performed in non-reduction conditions, 200 μL rAra h 1 was added by 6 μL MTG, then the mixture was heated at different temperatures (range: 40–60 °C) for varied times (range: 1–5 h).

For the cross-linking performed in reduced condition: rAra h 1 (200 μL) was added by DTT at different concentrations (range: 25–175 μg/mL), then the mixture was heated at 40 °C for one hour. After that, 6 μL MTG was added to each mixture, and then the samples were heated at 40 °C for 5 h to induce the cross-linking. After the MTG catalyzed reaction, the ionic salts in all the samples were removed by dialysis. Non-processed rAra h 1 was used as a control. After the reaction, products were stored at −80 °C until use.

### 2.4. Determination of Structural Alterations

#### 2.4.1. Polyacrylamide Gel Electrophoresis (PAGE)

The molecular weight and the charge interaction in the buildup of the protein polymers were monitored by Native and dodecyl sulfate, sodium salt (SDS)-Polyacrylamide gel electrophoresis (SDS-PAGE). The methods from Kiewiet were modified as follows [29]: protein samples (1.0 mg/mL) were mixed with loading buffer and denatured at 100 °C for 5 min. Electrophoresis was performed at 110 V for 80 min. After being stained by Coomassie Brilliant Blue R-250 for 40 min, the gels were the bleached overnight as described earlier [17]. Electrophoresis results were collected and analyzed by gel imaging system (BIO-RAD GelDoc 2000, CA, USA)

#### 2.4.2. Intrinsic Fluorescence Spectroscopy

After being loaded at a concentration of 1.0 mg/mL, the protein samples were analyzed by a Dual-FL fluorescence spectrophotometer (HORIBA, Kyoto, Japan). The excitation wavelength was set as 280 nm, and scanning intervals and slit width were set as described before [16]. Supporting software (Aqualog DualFL, HORIBA, Kyoto, Japan) was used to monitor the maximum emission wavelength.

#### 2.4.3. Dynamic Light Scattering

The protein size of rAra h 1 before and after modification was measured by DynaproNanoStar DLS machine (WYATT, Santa Barbara, CA, USA). Samples were analyzed three times, and the results were presented as intensity by size distribution.

#### 2.4.4. Determination of the Secondary Bonds

Methods from Tan [30] and Rao [31] were modified as follows: protein samples were lyophilized first, and each protein sample (1 g) was added by a different reagent (5 mL) to eliminate certain kinds of bonds: (1) 0.05 mol/L NaCl, (2) 0.6 mol/L NaCl, (3) 0.6 mol/L NaCl + 1.5 mol/L urea, (4) 0.6 mol/L NaCl + 8 mol/L urea. After reacting at 4 °C for 1 h, the mixtures were centrifuged at 20,000 g for 25 min. BCA assay was then used to define the protein content in supernatants. The ionic bonds were presented as the protein content difference between (1) and (2). The hydrogen bonds were the protein content difference between (3) and (2). The hydrophobic interactions were measured as the protein content difference between (4) and (3).

#### 2.4.5. Surface Hydrophobicity (H0) Measurement

The method reported by Mondoulet [32] was used in this part of the experiment. In short, samples were diluted to different concentrations as described earlier [33]. Protein samples (4 mL) were then mixed with ANS (8 mmol/L, 20 μL) and reacted in the dark for 15 min. The fluorescence intensity was detected by a F2500 fluorescence spectrometer (Hitachi, Tokyo, Japan), the excitation and emission wavelength were set as 390 and 470 nm, respectively. The surface hydrophobicity index (H0) was presented as the initial slope of fluorescence intensity versus protein concentration plot.

#### 2.4.6. Analysis of Protein Secondary Structures

As described before [17], protein samples (1.0 mg/mL) were loaded to a Chirascan spectroscope (Applied Photophysics Ltd., Surrey, UK). The scanning interval was set between 190 and 250 nm, with a scanning speed of 500 nm/min. After being analyzed by CDNN software package (Applied Photophysics Ltd., Surrey, UK), the content of different kinds of secondary structures were calculated.

#### 2.4.7. Spatial Structure Analysis

The spatial structure of Ara h 1 was built on the PyMOL soft [34] using Ara h 1 (PDB database, [35]) as a model. The information reported by Chruszcz [36] also helped in the modeling process. The linear epitopes and possible cross-linking sites located in allergic epitopes were marked in different colors on the monomer and trimer structure of Ara h 1.

### 2.5. Immunoreactivity Assessment

#### 2.5.1. Enzyme Linked Immunosorbent Assay (ELISA)

Protein samples were diluted to 10 μg/mL and added to a 96-well plate, as described [16]. After being incubated overnight, a blocking buffer (the recipe was described before [16]) was added and incubated with samples at 37 °C for one hour [37]. The plate was then added to the pooled patients’ serum (1:10 diluted in blocking buffer, *v/v*) at a volume of 100 μL per well. After being washed five times, the peroxidase-labeled goat anti-human IgE dissolved in the blocking buffer (1:5000, *v:v*) (100 μL per well) was loaded and the incubation lasted for 1 h at 37 °C. TMB (3,3,5,5-tetramethyl benzidine) was added to each well at a volume of 100 μL per well, and reaction lasted for 15 min. The reaction was ended by H_2_SO_4_ (2 mol/L) and the optical intensity was read at 450 nm.

#### 2.5.2. Immunoblotting

Immunoblotting was used to evaluated the IgG-binding ability of protein samples. According to Hurlburt et al. [38], protein from the SDS-PAGE gels was transferred to a nitrocellulose membrane. The tris buffered saline tween (TBST) buffer (the recipe was described before [16]) was utilized as the blocking buffer and the membrane was blocked at 37 °C for 2 h. Rabbit anti-Ara h 1 antibody (diluted in the blocking buffer by (1:5000 (*v/v*)) was used to bind proteins located in the membrane at 4 °C for 12 h. After washing, goat anti-rabbit IgG-HRP was utilized as secondary antibody to incubate the membrane for 2 h at 37 °C. Enhanced chemiluminescence (ECL) detection kit was used to visualize the results. The results were photographed and analyzed in gel imaging system (Tanon 5200Multi, Shanghai, China).

#### 2.5.3. Degranulation Test

Rat basophilic leukemia cell (RBL-2H3) was purchased from Kebai Biology Company (Nanjing, China). Cells were cultured as described before [13]. RBL-2H3 is a basophilic leukemia cell line isolated from Wistar rat basophilic cells, thus it can only be sensitized by murine serum. The detailed methods for the animal sera preparation can be found in the supplementary documents.

Briefly, RBL-2H3 cell were plated in 96-well plates at 1.5 × 10^5^ cells/mL (50 μL per well). After being incubated by murine serum overnight, the cells were sensitized with protein samples (at different concentrations) for 45 min. The antigenicity of different samples was measured by detecting the β-hexosaminidase release, and expressed as percentage of the total β-hexosaminidase content. Cells were lysed by 1% Triton X-100 and the β-hexosaminidase release were measured by using 4-nitrophenyl-N-acetyl-β-D-glucosaminide as described [13].

#### 2.5.4. Statistical Analysis

Each experiment was repeated at least three times. Statistical analysis was performed by SPSS Software (v15.0, SPSS Inc., Chicago, IL, USA). Difference was considered statistically significant when *p*-values were less than 0.05 in one-way Analysis of Variance (ANOVA) test.

## 3. Results and Discussion

### 3.1. Preparation of Cross-Linked rAra h 1

#### 3.1.1. Influence of Protein Concentration on the rAra h 1 Aggregation

Researchers have expressed the core region of Ara h 1 and found that the sensitivity of Ara h 1 is preserved in the recombinant protein [36,39]. However, it cannot be neglected that the linear epitopes of Ara h 1 start from 25 and end in 606. The core region (170–586) cannot cover the whole allergic area of the protein. There will be nine linear epitopes missing if we only express the core region of Ara h 1. In order to thoroughly explore the sensitivity changes, as well as detect the effect of cross-linking on the entire structure of rAra h 1, here, we expressed the full-length Ara h 1 in *Escherichia coli BL21(DE3) pLysS*. According to our results, the content of rAra h 1 is relatively high and the protocol we used to purify the protein is effective [17]. The amino acid sequence and secondary structure of rAra h 1 are similar, with natural Ara h 1. The difference in immunoreactivity between recombinant and natural Ara h 1 is not significant [17], for the linear and conformational epitopes are the dominant factors which influence the allergenicity of Ara h 1.

**Figure 1:**
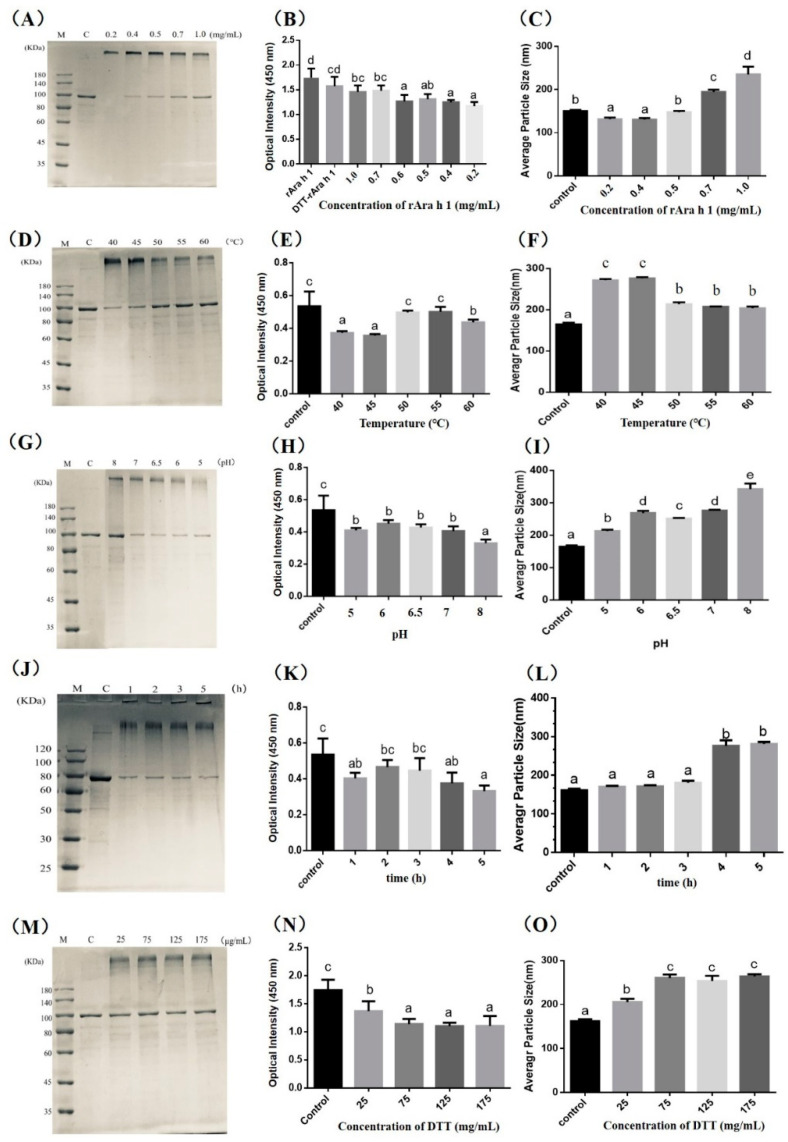
Influence of different reaction parameters on the molecular weight, IgE-binding capacity and particle size of cross-linked rAra h 1. (**A**,**D**,**G**,**J**,**M**) represents the effect of substrate concentration, temperature, pH, reaction time and DL-dithiothreitol (DTT) concentration on the molecular weight of rAra h 1 separately. (**B**,**E**,**H**,**K**,**N**) represents the effect of substrate concentration, temperature, pH, reaction time and DTT concentration on the IgE-binding efficiency of rAra h 1 separately. (**C**,**F**,**I**,**L**,**O**) represents the effect of substrate concentration, temperature, pH, reaction time and DTT concentration on the average particle size of rAra h 1 separately. The letters a, b, c, d and e represent for the significant difference between results. M represent for the protein marker, and C represent for the control group.

As seen in Figure 1A,D,G,J,M, the molecular weight of untreated rAra h 1 is 110 kDa. Compared with natural Ara h 1 (MW: 63.5 kDa), the molecular weight of the rAra h 1 (molecular weight: 110 kDa) was increased by 46.5 kDa. This is mainly due to the addition of Trx-tag, His-tag, S-tag and the other nine cleavage sites existing in the pET-32a vector. There are 1162 base pairs added to the DNA sequence after the promoter, and the additional base pairs in the recombinant plasmids lead to the production of an extra 388 amino acids, which caused a 46.5 kDa increase in the molecular weight of rAra h 1.

The addition of MTG significantly promoted the aggregation of rAra h 1. As shown in Figure 1A, cross-linking between protein molecules could happen when the protein concentration is relatively low (0.2 mg/mL). When the protein concentration increased (0.4–1.0 mg/mL), the amount of polymer did not show any significant increase. Aggregation could decrease the allergenicity of rAra h 1, as seen in Figure 1B. The IgE-binding capacity decreased to the minimum among the tested samples when the protein concentration was lower than 0.4 mg/mL; this may due to the decline in allergen content, or the shielded epitopes formed during the aggregation.

MTG has been reported to modify proteins by catalyzing acyl transfer reactions, crosslinking, and deamidation [40]. Briefly, MTG could catalyze acyl transfer reaction between γ-carboxyamide group of a glutamine residue and a primary amine. A cross-linking reaction occurs when the ε-amino group of a lysine residue in a protein acts as an acyl acceptor. Deamidation would happen when the water becomes the acyl acceptor; glutamine residues could be converted to glutamic acid residues at this time [41]. The structural characteristics of products mainly depend on the specific reaction conditions, like substrate concentration, temperatures, and pH et al. According to Wu’s research [23], in MTG-catalyzed aggregation, intermolecular crosslinking was more likely to happen when the concentration of reduced Ara h 2 was higher than 0.7 mg/mL. Comparably, intramolecular crosslinking was more likely to be induced when the concentration of reduced Ara h 2 ranged from 0.2 to 0.4 mg/mL. For now, there are no restrictions on MTG addition in the food industry. Some researchers produced aggregated proteins with a significantly reduced allergenicity by adding the MTG at 30 U/g [24] and 50 U/g [42]. According to these studies, we set the rAra h 1 concentrations as 0.2, 0.4, 0.5, 0.7 and 1.0 mg/mL, which correspond with MTG addition of 30, 37.5, 50, 60, 75, 150 U/g.

As seen in Figure 1C, the average particle size decreased when the protein concentration was lower than 0.4 mg/mL, illustrating the possibility of intramolecular crosslink or deamidation happened inside of the protein molecules. This result is consistent with the former studies [23,24]. The significant increase in particle size occurred when the concentration of rAra h 1 was higher than 0.7 mg/mL, illustrating the intermolecular crosslinks happened during MTG-catalyzed reactions. To produce protein aggregates with lower sensitivity, the concentration of rAra h 1 was set as 1.0 mg/mL in the following experiments.

#### 3.1.2. Effect of Reaction Conditions (Temperature, pH, and Time) on the rAra h 1 Aggregation

The optimal working temperature for MTG is between 50 and 55 °C, and MTG will be inactivated within a few minutes when the temperature is higher than 75 °C [40]. We performed the experiments in the temperature range from 40 to 60 °C, aiming to identify the optimum temperature for cross-linking. As seen in Figure 1D, the content of polymers produced in 40 and 45 °C is significantly higher compared with control. Corresponding with this, the IgE-binding capacity reached the minimum in the mentioned two samples in Figure 1E, and the average particle size of products reached the maximum in 40 and 45 °C groups in Figure 1F. Although rAra h 1 could be cross-linked in all tested temperatures, aggregates with decreased antigenicity were easier to be produced in 40 and 45 °C. According to this, we then used 40 °C in the following experiments.

MTG was active when the pH ranged from 5.0 to 8.0 [22]. Polymers could be produced in all tested pH conditions (Figure 1G). The content of both polymers and monomers reached the bottom when the pH was 5.0, which is due to the decrease in the solubility of rAra h 1 when pH nears its isoelectric point (4.55). An approximately 33.33% decrease in IgE-binding efficiency happened as pH ranged from 5.0 to 7.0 compared with control (Figure 1H). However, the significant decrease (42.38%) was presented in the pH 8.0 group. To explain this result, we have to know that Ara h 1 is an acidic protein. In strong alkaline conditions, hydroxide ions would be bound to the protein surface by hydrogen bonds, thus decreasing the surface hydrophobic index. Meanwhile, lysine and arginine were blocked. It is reported that protein polymers with a reduced surface hydrophobic index are difficult to bind with IgE [16,17]. Consistent with the immunoreactivity change in Figure 1H, the particle size of aggregates produced in pH 8 reached the top among all tested samples (Figure 1I). Besides, the pH value influenced the particle size significantly, for there is, separately, a 20.78%, 54.50%, 39.89%, 55.01% and 90.85% increase in the pH 5, 6, 6.5, 7 and 8 groups (Figure 1I). According to the results, we then used pH 8 in the following experiments.

According to the research which used MTG-catalyzed reactions to lower the antigenicity of peanut proteins [23,24], the reaction time was normally set between 4 and 5 h. Here, we tested the influence of different reaction times (1–5 h) on the structure and antigenicity of cross-linked rAra h 1. As shown in Figure 1J, the content of monomer decreased significantly when the reaction time reached 1 h. However, the content of the high-molecular-weight polymer did not increase along with the time extension. The IgE-binding capacity significantly reduced when the protein was cross-linked, and the bond value reached the bottom at 5 h in Figure 1K. The results in Figure 1L showed that the molecular size increased with the time extension. To produce aggregated rAra h 1 with reduced antigenicity, we then used 5 h as the reaction time in the following experiments.

#### 3.1.3. Effect of Reduction Extent on the rAra h 1 Cross-Linking

DTT could destabilize peanut allergens and thus expose the glutamine residues. Besides, as a common deoxidizer, DTT could neutralize various oxidants in the reaction system and protect the cysteine residues from oxidation, thereby accelerating the enzyme cross-linking reactions. After being treated with DTT, Ara h 2 could be digested by pepsin easily, and the immunoreactivity decreased too [24]. According to these results, here, we tested different concentrations of DTT to find the optimal condition.

As seen in Figure 1M, when the concentration of DTT increased, the content of polymers did not show a significant increase. Compared with the control, the IgE-binding capacity decreased obviously when DTT was added at 25 and 75 μg/mL (Figure 1N). However, the bond value did not significantly decrease when DTT ranged from 75 to 175 μg/mL. Corresponding with this, the average particle size did not show an increase when the DTT ranged at the same scale. To produce aggregated rAra h 1 with reduced antigenicity, we then used 75 μg/mL as the DTT concentration in the following experiments.

To explore the influence of specific procedures of MTG-catalyzed cross-linking on the immunoreactivity of aggregated rAra h 1, five samples including non-processed rAra h 1 (named rAra h 1), rAra h 1 heated at 40 °C for 5 h (named heated), rAra h 1 reduced by DTT (named DTT), rAra h 1 cross-linked by MTG directly (named MTG), and rAra h 1 reduced by DTT and then cross-linked by MTG (named DTT + MTG) were selected to be structurally studied. The immunoreactivity of mentioned samples was also measured both by immunoblots and degranulation tests.

### 3.2. Structural Changes in Polymerized rAra h 1

#### 3.2.1. Intrinsic Fluorescence and Molecular Size

**Figure 2:**
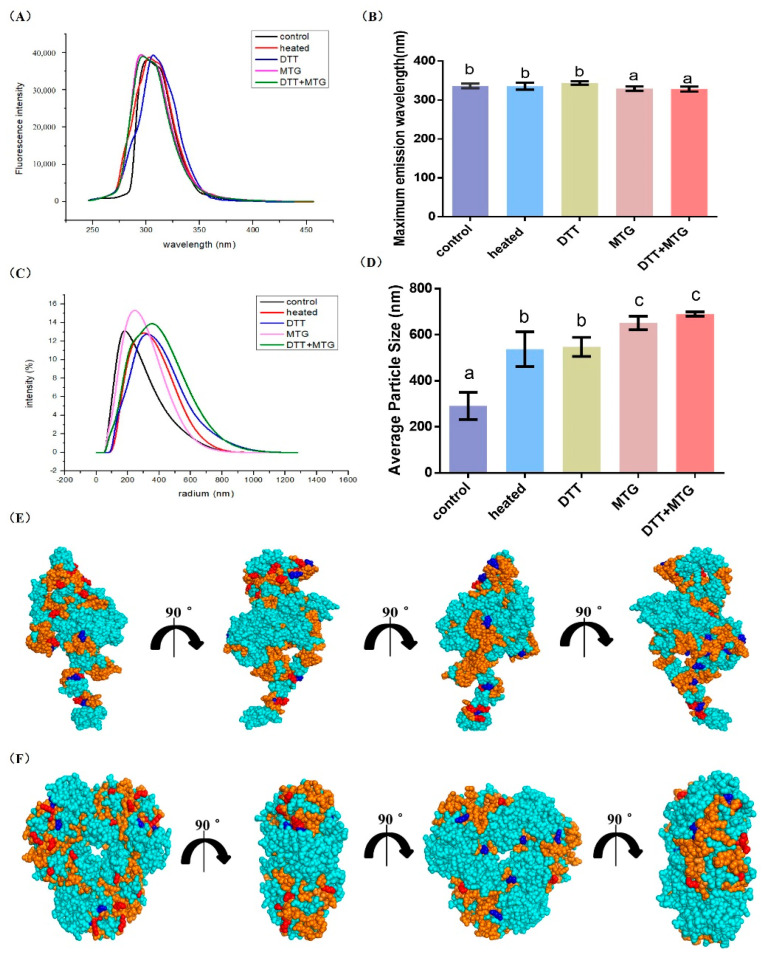
Structure features of rAra h 1 before and after modification. (**A**) Fluorescence spectrum plots, (**B**) Maximum emission wavelength, (**C**) Dynamic light scattering, and (**D**) Average particle size of protein samples. Control: non-processed rAra h 1, heated: rAra h 1 heated at 40 °C for 5 h, DTT: rAra h 1 reduced by DTT, microbial transglutaminase (MTG): rAra h 1 cross-linked by MTG, DTT + MTG: rAra h 1 reduced by DTT first, and then cross-linked by MTG. The letters a, b, and c represent for the significant difference between results. (**E**,**F**) are the globular presentations of linear epitopes as well as possible cross-linking sites on the monomer and trimer of rAra h 1, respectively. The epitopes are marked in orange, Lys and Gln located in the epitopes are marked in red and blue separately.

As seen in Figure 2A,B, once MTG was added to the reaction system, the blue shifts (about 8 nm) in emission wavelength would happen in two samples, MTG-rAra h 1 and (DTT + MTG)-rAra h 1. From this result, it can be deducted that MTG catalyzed cross-linking can reduce the electron density around tryptophan, so peptides could not stretch freely, which thus caused a relatively tight structure in polymers.

According to Figure 2C,D, the molecular size was increased in modified protein. The significant increase existed in two samples (MTG-rAra h 1 and (DTT + MTG)-rAra h 1), by increasing the number from 251.162 to 651.388 and 689.847, respectively (Figure 2D). Compared with these two, relatively mild increases could be found in heated and reduced rAra h 1. The number lifted by 246.345 and 256.178, respectively. It can be seen that the participation of MTG could effectively cross-link rAra h 1 to a relatively high extent.

The spatial structure of rAra h 1 was modeled and presented in Figure 2E,F: the 23 linear epitopes were marked orange, and Lys and Gln residues located in the epitopes were marked red and blue, respectively. As seen, 17 linear epitopes were exposed on the protein surface. Among them, Lys residues were contained in 10 epitopes, and Gln were contained in seven epitopes. As the possible cross-link sites for MTG-catalyzed reaction, Lys and Gln could prompt the cross-linking between epitopes, and thus cause a reduced immunoreactivity of rAra h 1.

#### 3.2.2. Aggregation Mode, Surface Hydrophobicity and Chemical Bonds

Covalent cross-linking and non-covalent interactions were normally involved in protein aggregations. For covalent cross-linking, disulfide bonds were normally needed to form polymers and help to maintain the stability of aggregates. Non-covalent interactions (containing hydrogen bonds, salt bridge, and hydrophobic interactions) usually participated in the aggregation process, and are easier to disrupt compared with covalent bonds [31]. The charge interactions in the construction of the polymers were analyzed by comparing the native PAGE results and SDS-PAGE gels [29]. SDS-PAGE was performed in the presence of DTT, which was used to break sulfide bonds. Besides, SDS is an anionic detergent as well as a cosolvent, which could break the hydrogen bonds between molecules, thus unfolding the molecule, and further destroying the secondary and tertiary structure of the protein particles.

**Figure 3:**
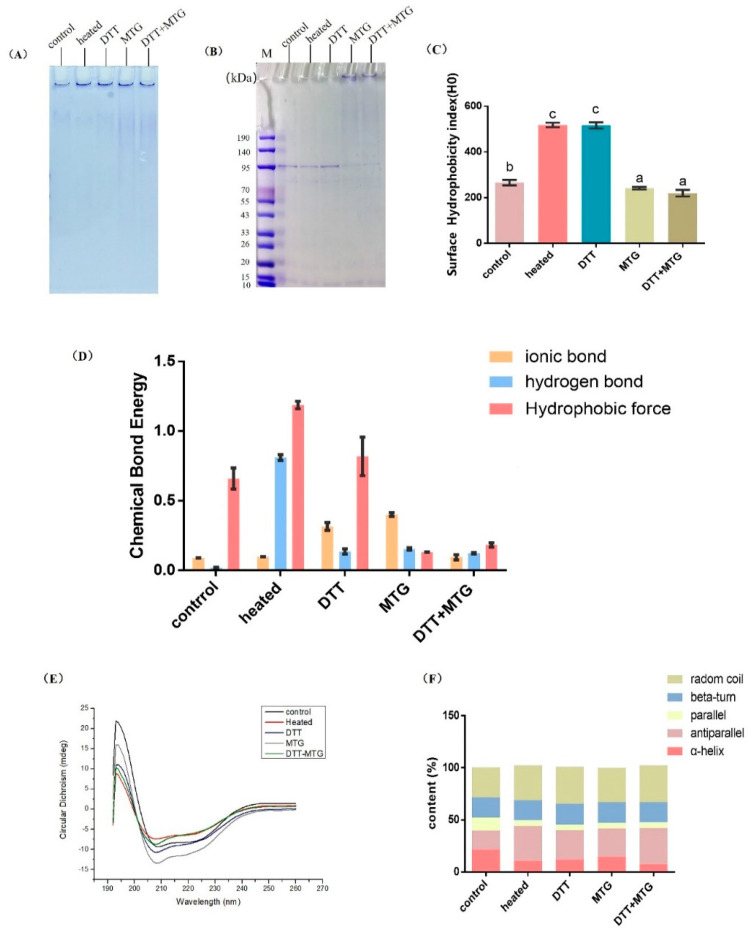
Aggregation mode, chemical bonds, and secondary structural features of cross-linked rAra h 1. (**A**,**B**) are the Native-PAGE and SDS-PAGE separately. (**C**) Surface hydrophobic index, (**D**) Content of different chemical bonds, (**E**) Circular dichroism spectrum plots, and (**F**) The proportion of different secondary structures of rAra h 1 before and after modification. Control: non-treated rAra h 1, heated: rAra h 1 heated at 40 °C for 5 h, DTT: rAra h 1 reduced by DTT, MTG: rAra h 1 cross-linked by MTG, DTT + MTG: rAra h 1 reduced by DTT first and then cross-linked by MTG. The letters a, b, and c represent for the significant difference between results.

According to the results, rAra h, 1s after heating and reducing, was assembled in the loading wells in the Native-PAGE pattern (Figure 3A, lane 1 and 2), but only monomers appeared in the SDS-PAGE (Figure 3B, lane 1 and 2), illustrating that hydrogen bonds and disulfide bonds participated in the polymerization in these two samples. Comparably, rAra h 1 cross-linked by MTG was more stable, for there were both monomers and polymers in the SDS-PAGE pattern (Figure 3B, lane 3 and 4). It shows that ε-(γ-glutamine) lysine isopeptide bonds were formed in the polymers produced by MTG and DTT + MTG treatment, which then caused a relatively compact and stable structure of aggregates.

As seen in Figure 3C, the surface hydrophobicity index increased in heated rAra h 1 and DTT-reduced rAra h 1 when compared with the control, indicating that there were more hydrophobic groups outside of the mentioned protein samples, and the protein structure was correspondingly looser. Compared with the control, the index was mildly decreased by 28.52% and 28.98% in MTG- and DTT + MTG-treated samples separately (Figure 3C); this is attributed to the fact that the protein structure was compact in those two samples, which is consistent with the result in Figure 2B.

As seen in Figure 3D, the content of the ionic bond increased significantly in DTT- and MTG-treated rAra h 1s, indicating that ionic bonds have promoted molecular aggregation and improved the stability of the polymers in these two groups. It is worth noticing that the content of hydrogen bonds increased dramatically in heated rAra h 1 compared with control, illustrating that the polymers induced by heating were stabilized mainly by hydrogen bonds; this result is consistent with Figure 3A (lane 1).

The high content of hydrophobic force could be found in control, heated and DTT-reduced rAra h 1, illustrating that the hydrophobic residues were more exposed to the protein surface, thus causing a relatively looser structure. Besides, the aromatic side chains located in the lumen of rAra h 1 molecules would flip to the protein surface during the heating and reduction process, and then caused an increased hydrophobic force; this is consistent with results in Figure 3C. The hydrophobic force decreased significantly when rAra h 1 was treated with MTG and DTT + MTG, which indicated that an inner-locking structure was probably formed in these two groups. At last, the ionic bonds, hydrogen bonds, and hydrophobic force were kept at a relatively low content in rAra h 1 treated with DTT + MTG, indicating that the polymers formed in this group were mainly stabilized by covalent cross-linking; this is consistent with the result in Figure 3B.

#### 3.2.3. CD Spectra

According to Figure 3E, typical α-helixes existed in all samples, for positive peaks around 192–195 nm and negative peaks at 208 nm appeared in each curve. For non-processed rAra h 1, the peak in 195 nm is the highest among the tested samples, indicating the highest content of α-helixes in five samples. The content of α-helixes, β-turns, random coils and antiparallel were analyzed by CDNN software (Figure 3F). As seen, the percentage of α-helixes declined in heated, DTT-reduced, MTG-treated and DTT + MTG-treated samples by 68.98%, 44.91%, 31.48% and 64.81%, respectively. Nevertheless, there was a 23.34%, 16.38%, 14.98% and 23.34% increase in the content of antiparallel in heated, DTT-reduced, MTG-treated and DTT + MTG-treated rAra h 1. According to these results, the protein secondary structure changed to a disordered state in all the treated samples, and re-folding, re-curling and re-swing of protein molecules occurred in the cross-linking process. Conservative secondary structures, like α-helixes and β-sheets, could protect the linear epitopes from disrupting. The decreased content of α-helixes could be a reason for the reduction in immunoreactivity in treated samples.

### 3.3. Effect of Cross-Linking on the Immunoreactivity

#### 3.3.1. Immunoblots and ELISA Analysis

**Figure 4:**
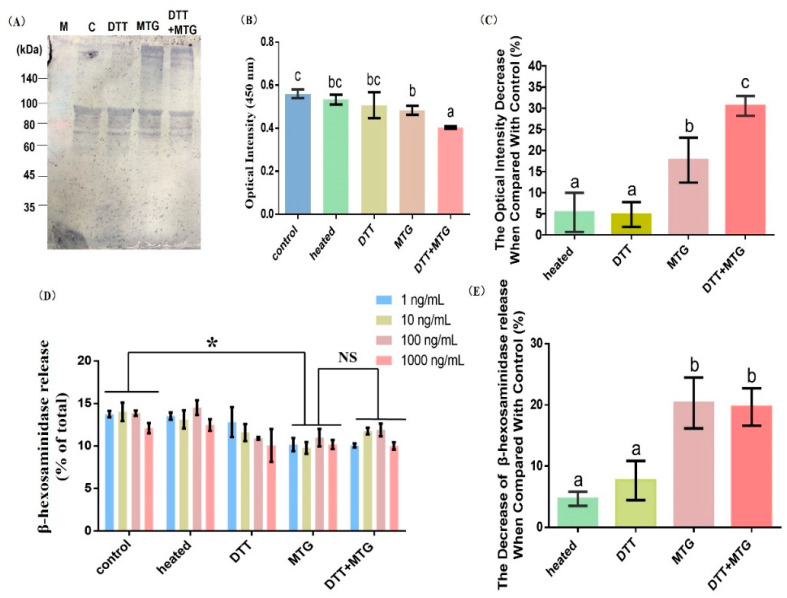
Immunoreactivity changes of rAra h 1 before and after modification. (**A**) Immunoblots by using rabbit anti-Ara h 1 antibody. (**B**) ELISA results by using pooled patients’ sera. (**C**) The decrease in IgE-binding efficiency in different samples when compared with the control group. (**D**) β-hexosaminidase release of cells stimulated by protein samples. (**E**) The decrease in β-hexosaminidase release in different samples when compared with control group. M: protein marker, C or Control: rAra h 1 before modification, heated: rAra h 1 heated at 40 °C for 5 h, DTT: rAra h 1 reduced by DTT, MTG: rAra h 1 cross-linked by MTG, DTT + MTG: rAra h 1 reduced by DTT first and then cross-linked by MTG. Results were considered significantly different from the control when * *p* < 0.05 in the one-way ANOVA test. NS, not significant. The letters a, b, and c represent for the significant difference between results.

The only monomer bond with rabbit IgG antibody appeared in the first two lanes in the immunoblot pattern (Figure 4A,C and DTT). Fainter bands around 80 kDa were also bound with antibodies; this may due to the low specificity of the rabbit-derived rAra h 1 antibody, or because of the degradation of rAra h 1 during the experiment. Comparably, the addition of MTG in samples promoted aggregation and induced high molecular polymers (>180 kDa), which could bind with rabbit IgG antibody (Figure 4A, MTG and DTT + MTG). According to the ELISA results, heating and DTT treatment cannot significantly reduce the sensitization of rAra h 1(Figure 4B,C). When MTG is added to the reaction system, aggregates with reduced antigenicity were produced. Furthermore, the IgE-binding capacity decreased to its minimum when rAra h 1 was reduced by DTT first and then cross-linked by MTG.

#### 3.3.2. β-hexosaminidase Release

As seen in Figure 4D, different concentrations of rAra h 1 were regarded as repeats, and we performed the statistical analysis. The degranulation rate decreased when rAra h 1 was cross-linked by MTG (Figure 4D,E, MTG and DTT + MTG). Besides, there is no difference between MTG and DTT + MTG groups (Figure 4D,E). These results are consistent with Figure 4B. It has been reported that MTG-catalyzed cross-linking could induce irreversible structural changes and thus cause a loss of conformational and linear epitopes of the allergen [24]. Upon unfolding, the tertiary and quaternary structures were disrupted, which could explain the reason for the reduced degranulation rate in cross-linked rAra h 1 (Figure 4D, MTG and DTT + MTG).

Different procedures in food processing have various effects on the antigenicity of allergens. According to our results, heating and reducing cannot decrease the immunoreactivity significantly, but the MTG-catalyzed cross-linking could modestly but significantly reduce the antigenicity of rAra h 1. This can be attributed to the increased steric hindrance and destructed epitopes.

Ara h 1 is one of the major allergens in peanut, and developing effective methods used for lower its sensitivity is essential in the desensitizing research. In this study, we analyzed the effect of cross-linking on the structure and allergenicity changes in rAra h 1 step by step, aiming to find the key factors affecting the antigenicity decrease in rAra h 1. The secondary structural changes, tertiary structural alterations, quaternary structural features and allergenicity changes in rAra h 1 during the cross-linking were detected. The results from this research could provide a more realistic picture of the application of desensitizing processing. For example, the reaction conditions for cross-linking have been tested and recommended by our research, and now the same condition could be used to treat the entire peanut protein and explore the desensitization effects. Besides, in this research we found that different protein structures were influenced in various processing steps. These results could provide new information when we try to induce specific structural changes in the allergens in future studies. At last, cross-linked rAra h 1, which contains a lowered sensitivity, could be used as a vaccine in oral immunotherapy for peanut-allergic patients.

## 4. Conclusions

Polymers with lowered allergenicity were formed when rAra h 1 was cross-linked by MTG. The optimal conditions for the cross-linking are 1.0 mg/mL. rAra h 1 was firstly reduced by DTT (75 μg/mL) at 40 °C for 1 h. Then, the mixture was then added to MTG at 30 U/g (pH 8), and reacted at 40 °C for 5 h. The polymers formed during cross-linking have different structural features at different reaction stages. Hydrogen bonds and disulfide bonds were the main molecular forces in polymers treated by heat and DTT. However, ε-(γ-glutamyl) lysine isopeptide bonds were formed during MTG-catalyzed cross-linking, which leads to polymers with a relatively compact and stable structure. Although the cross-linked product of rAra h 1 could bind with antibodies, its sensitivity was significantly lower than that of untreated rAra h 1. This is due to the fact that the content of the conserved structure in secondary structures was reduced and then led to a loss of protection of linear epitopes. Besides, the decreased surface hydrophobic index and increased steric hindrance of the cross-linked rAra h 1 made it more difficult to bind with IgE, and thus hindered the progress of subsequent allergic reactions.

## Supplementary Materials

The ethical approval is available online at https://www.mdpi.com/2304-8158/9/10/1508/s1. Table S1: Clinical information of peanut allergic patients. Supplementary file: The detailed methods for the animal sera preparation.

## References

[1] Sicherer S.H., Sampson H.A. (2018). Food allergy: A review and update on epidemiology, pathogenesis, diagnosis, prevention, and management. J. Allergy Clin. Immunol..

[2] Wang Y., Ni S., Wang C., Li X., Fu L. (2019). Cross-linking of shrimp tropomyosin catalyzed by transglutaminase and tyrosinase produces hypoallergens for potential immunotherapy. Food Funct..

[3] Zhou Y., Wang J.S., Yang X.J., Lin D.H., Gao Y.F., Su Y.J., Yang S., Zhang Y.J., Zheng J.J. (2013). Peanut Allergy, Allergen Composition, and Methods of Reducing Allergenicity: A Review. Int. J. Food Sci..

[4] Jones S.M., Sicherer S.H., Burks A.W., Leung D.Y.M., Lindblad R.W., Dawson P., Henning A.K., Berin M.C., Chiang D., Vickery B.P. (2017). Epicutaneous immunotherapy for the treatment of peanut allergy in children and young adults. J. Allergy Clin. Immunol..

[5] Burks W., Sampson H.A., Bannon G.A. (1998). Peanut allergens. Allergy.

[6] Blanc F., Vissers Y.M., Adel-Patient K., Rigby N.M., Mackie A.R., Gunning A.P., Wellner N.K., Skov P.S., Przybylski-Nicaise L., Ballmer-Weber B. (2011). Boiling peanut Ara h 1 results in the formation of aggregates with reduced allergenicity. Mol. Nutr. Food Res..

[7] Ramesh M., Yuenyongviwat A., Konstantinou G.N., Lieberman J., Pascal M., Masilamani M., Sampson H.A. (2016). Peanut T-cell epitope discovery: Ara h 1. J. Allergy Clin. Immunol..

[8] Maleki S.J., Kopper R.A., Shin D.S., Park C.W., Compadre C.M., Sampson H., Burks A.W., Bannon G.A. (2000). Structure of the major peanut allergen Ara h 1 may protect IgE-binding epitopes from degradation. J. Immunol..

[9] Maleki S. (2014). The molecular effects of processing on the peanut allergens. Clin. Transl. Allergy.

[10] Li H., Yu J., Ahmedna M., Goktepe I. (2013). Reduction of major peanut allergens Ara h 1 and Ara h 2, in roasted peanuts by ultrasound assisted enzymatic treatment. Food Chem..

[11] López E., Cuadrado C., Burban C. (2012). Effects of autoclaving and high pressure on allergenicity of hazelnut proteins. J. Clin. Bioinform..

[12] Cuadrado C., Cheng H., Sanchiz A., Ballesteros I., Easson M., Grimm C.C., Dieguez M.C., Linacero R., Burbano C., Maleki S.J. (2018). Influence of enzymatic hydrolysis on the allergenic reactivity of processed cashew and pistachio. Food Chem..

[13] Rao H., Chen C., Tian Y., Li Y., Gao Y., Tao S., Xue W. (2018). Germination results in reduced allergenicity of peanut by degradation of allergens and resveratrol enrichment. Innov. Food Sci. Emerg. Technol..

[14] Fu W., Xue W., Liu C., Tian Y., Zhu Z. (2020). Screening of Lactic Acid Bacteria and Yeasts from Sourdough as Starter Cultures for Reduced Allergenicity Wheat Products. Foods.

[15] Vanga S.K., Singh A., Raghavan V. (2015). Effect of thermal and electric field treatment on the conformation of Ara h 6 peanut protein allergen. Innov. Food Sci. Emerg. Technol..

[16] Tian Y., Liu C., Zhang K., Tao S., Xue W. (2020). Glycosylation between recombinant peanut protein Ara h 1 and glucosamine could decrease the allergenicity due to the protein aggregation. LWT Food Sci. Technol..

[17] Tian Y., Rao H., Tao S., Xue W.T. (2018). Effect of boiling on the structure and immunoreactivity of recombinant peanut protein Ara h 1. Food Agric. Immunol..

[18] Comstock S.S., Maleki S.J., Teuber S.S., Thierry C. (2016). Boiling and Frying Peanuts Decreases Soluble Peanut (*Arachis hypogaea*) Allergens Ara h 1 and Ara h 2 But Does not Generate Hypoallergenic Peanuts. PLoS ONE.

[19] Tian Y., Rao H., Fu W., Tao S., Xue W.-T. (2019). Effect of digestion on the immunoreactivity and proinflammatory properties of recombinant peanut allergen Ara h 1. Food Agric. Immunol..

[20] Novak N., Maleki S.J., Cuadrado C., Crespo J.F., Cabanillas B. (2020). Interaction of Monocyte-Derived Dendritic Cells with Ara h 2 from Raw and Roasted Peanuts. Foods.

[21] Kuraishi C., Nakagoshi H., Tanno H., Tanaka H. (2000). Application of transglutaminase for food processing. Hydrocolloids.

[22] Chang X., Wu Z., Zhao R., Zhang Y., Li X., Yang A., Tong P., Chen H. (2018). Analysis on MTGase catalysed cross-linked products of Ara h 2: Structure and immunoreactivity. Food Agric. Immunol..

[23] Wu Z., Zhao R., Ren L., Li X., Yang A., Tong P., Chen H. (2017). Modification of the reaction system of Ara h 2 catalyzed by MTGase: Products and reaction conditions analysis. J. Food Process. Preserv..

[24] Wu Z., Lian J., Zhao R., Li K., Li X., Yang A., Tong P., Chen H. (2017). Ara h 2 cross-linking catalyzed by MTGase decreases its allergenicity. Food Funct..

[25] Tong P., Chen S., Gao J., Li X., Wu Z., Yang A., Yuan J., Chen H. (2017). Caffeic acid-assisted cross-linking catalyzed by polyphenol oxidase decreases the allergenicity of ovalbumin in a Balb/c mouse model. Food Chem. Toxicol..

[26] Tian S., Ma J., Ahmed I., Lv L., Li Z., Lin H. (2019). Effect of tyrosinase-catalyzed crosslinking on the structure and allergenicity of turbot parvalbumin mediated by caffeic acid. J. Sci. Food Agric..

[27] Ahmed I., Lv L., Lin H., Li Z., Ma J., Guanzhi C., Sun L., Xu L. (2017). Effect of tyrosinase-aided crosslinking on the IgE binding potential and conformational structure of shrimp (*Metapenaeus ensis*) tropomyosin. Food Chem..

[28] Nagy E.D., Guo Y., Tang S., Bowers J.E., Okashah R.A., Taylor C.A., Zhang D., Khanal S., Heesacker A.F., Khalilian N. (2012). A high-density genetic map of Arachis duranensis, a diploid ancestor of cultivated peanut. BMC Genom..

[29] Kiewiet M.B., Dekkers R., Ulfman L.H., Groeneveld A., de Vos P., Fass M.M. (2018). Immunomodulating protein aggregates in soy and whey hydrolysates and their resistance to digestion in an in vitro infant gastrointestinal model: New insights in the mechanism of immunomodulatory hydrolysates. Food Funct..

[30] Tan F.J., Lai K.M., Hsu K.C. (2010). A Comparative Study on Physical Properties and Chemical Interactions of Gels from Tilapia Meat Pastes induced by Heat and Pressure. J. Texture Stud..

[31] Rao H., Tian Y., Tao S., Tang J., Li X., Xue W.T. (2016). Key factors affecting the immunoreactivity of roasted and boiled peanuts: Temperature and water. LWT Food Sci. Technol..

[32] Mondoulet L., Paty E., Drumare M.F., Ah-Leung S., Scheinmann P., Willemot R.M., Wal J.M., Bernard H. (2005). Influence of thermal processing on the allergenicity of peanut proteins. J. Agric. Food Chem..

[33] Tian Y., Rao H., Zhang K., Tao S., Xue W.T. (2018). Effects of different thermal processing methods on the structure and allergenicity of peanut allergen Ara h 1. Food Sci. Nutr..

[34] http://www.pymol.org/.

[35] http://www.rcsb.org/structure/3SMH.

[36] Chruszcz M., Maleki S.J., Majorek K.A., Demas M., Bublin M., Solberg R., Hurlburt B.K., Ruan S., Mattison C.P., Breiteneder H. (2011). Structural and immunologic characterization of Ara h 1, a major peanut allergen. J. Biol. Chem..

[37] Liu C., Tao S., Xue J., Zhang H., Xue W., Chen F. (2014). Identification and purification of a novel fish allergen from largemouth bass (*Micropterus salmoides*). Food Agric. Immunol..

[38] Hurlburt B.K., Mcbride J.K., Nesbit J.B., Ruan S., Maleki S.J. (2014). Purification of Recombinant Peanut Allergen Ara h 1 and Comparison of IgE Binding to the Natural Protein. Foods.

[39] Cabanos C., Tandang-Silvas M.R., Odijk V., Brostedt P., Tanaka A., Utsumi S., Maruyama N. (2010). Expression, purification, cross-reactivity and homology modeling of peanut profilin. Protein Expr. Purif..

[40] Nielsen P.M. (1995). Reactions and potential industrial applications of transglutaminase. Review of literature and patents. Food Biotechnol..

[41] Miwa N. (2020). Innovation in the food industry using microbial transglutaminase: Keys to success and future prospects. Anal. Biochem..

[42] Fangzhou Y., Ishfaq A., Liangtao L., Zhaojie L., Zhenxing L., Hong L., Hang L., Jinxia Z., Shenglan T., Jiaju M. (2018). Impacts of glycation and transglutaminase-catalyzed glycosylation with glucosamine on the conformational structure and allergenicity of bovine β-lactoglobulin. Food Funct..

