# Supplementary material for: Crosslinked Recombinant-Ara h 1 Catalyzed by Microbial Transglutaminase: Preparation, Structural Characterization and Allergic Assessment
Source: Foods. 2020 Oct 21;9(10):1508. doi: 10.3390/foods9101508 (PMC7590132; doi:10.3390/foods9101508)
Supplement: Supplementary file 1 [file foods-09-01508-s001.pdf]

Crosslinked Recombinant-Ara h 1 catalyzed by Microbial Transglutaminase: Preparation, Structural Characterization and Allergic Assessment

Yang Tian [1,2], Chenglong Liu [3], Wentong Xue [3*], and Zhongfu Wang [1,2]

1 College of Food Science and Technology, Northwest University, Xi'an, 710069, People's Republic of China;
2 Research Center for Glycobiology and Glycotechnology, College of Food Science and Technology, Northwest University, Xi'an, 710069, People's Republic of China;
3 College of Food Science and Nutritional Engineering, China Agricultural University, Beijing, 100083, People's Republic of China;
* Correspondence: xwt@cau.edu.cn;

Supplementary Material

1. Supplementary Method

Preparation of Animal sera

Four-week-old female Balb/C mice were purchased from Weitonglihua Co. (Beijing China). The animal experiment was approved by the Ethics Committee of China Agricultural University (NO. 2017001). During the experiment, animals were maintained on peanut free chow under specific pathogen-free conditions. All the animals were placed in a room maintained at 23±2°C, 50±10% relative humidity, 15 times of air changes per hour and 12 h light/dark cycle.

Mice were randomly divided into the control and allergic groups (17 mice per group). Members in control group were daily fed with 100 μL phosphate buffered saline (PBS, pH 7.4) by intragastric administration for 30 days. While mouse in allergic group were exposed by gavage to 5 mg peanut protein with 10 μg Cholera toxin (CT) in 100 μL PBS on days 7, 14, 21, 28, and 35 respectively. On day 42, the allergic group was challenged by 10 mg peanut protein with 10 μg CT. Blood was collected 30 min after the oral exposure to peanut or saline on days 35 and 42. Sera were pooled, aliquoted, and stored at -20 °C until use.

2. Supplementary Tables

Table S-1. Clinical information of peanut allergic patients

Patient ID	Total IgE CAP (kU/L)	Peanut sIgE CAP (kU/L)	Ara h 1 sIgE CAP (kU/L)	Clinical Manifestations
1	380	63	30.9	Throat tightness; angioedema; abdominal pain; nausea; Wheeze;
2	530	30.8	20.6	Oral/lip itching/tingling; Oral/lip swelling; Rhinitis; Conjunctivitis;
3	440	20	9.7	Angioedema; Urticaria; Light-headedness/dizziness; Abdominal pain/cramps;
4	150	10.1	8.7	Itching; Angioedema; Urticaria; Chest tightness; Anaphylaxis;
5	260	15.8	9.0	Diarrhea; Urticarial; Asthma;
6	408	15	6.8	Vomiting; Diarrhea;
7	414	20	12.0	Lip itching/tingling; Diarrhea;
8	308	15	12.7	Abdominal pain/cramps; Urticaria;

Patient ID	Total IgE CAP (kU/L)	Peanut sIgE CAP (kU/L)	Ara h 1 sIgE CAP (kU/L)	Clinical Manifestations
9	210	19	14.8	Vomiting; Itching; Angioedema;
10	138	22	6.2	Oral/lip swelling; Rhinitis; Abdominal pain;
11	180	31	8.8	Angioedema; abdominal pain; Oral/lip swelling;
12	360	28.8	12.7	Diarrhea; Oral/lip itching/tingling;

中国农业大学人体研究伦理委员会审查批件

批件号：2017001

项目名称	源于“老酵头”酵母菌及乳酸菌降低面团致敏性机制研究		
审查文件及版本日期	（一）《人体研究申请表及受试者知情同意书》；<br>（二）人体研究之前的整套研究资料摘要；<br>（三）人体研究方案；<br>（四）《食品类人体研究计划主持人执行承诺书》；<br>（五）主要研究者专业履历 并签名。		
申请人	薛文通	联系方式	13910615439
申请单位	中国农业大学食品科学与营养工程学院		
审批意见	☼ 同意<br>☐ 作必要修该后同意<br>☐ 不同意<br>☐ 终止或暂停已批准的试验		
意见和建议	本伦理委员会在对该申请项目提交各部分文件进行充分了解和讨论基础上，认为该项目设计合理，在保护受试者隐私、保证受试者安全和尊重受试者权益等方面采取的措施合理恰当。对该项目的执行提出建议如下：（1）与血清提供单位密切配合，保证每一份血清提供者都知情；（2）血清具有传播疾病的风险，保护血清接触者的安全；（3）向血清提供者告知本实验的结果。		
主任委员签字	罗云波		

中国农业大学人体研究伦理委员会（盖章）

中国农业大学人体研究伦理委员会 1100000129383

日　期：2017.3.9
